# Jieduan-Niwan Formula Reduces Liver Apoptosis in a Rat Model of Acute-on-Chronic Liver Failure by Regulating the E2F1-Mediated Intrinsic Apoptosis Pathway

**DOI:** 10.1155/2019/8108503

**Published:** 2019-11-11

**Authors:** Wenlong Yang, Yulin Hao, Weixin Hou, Xian Fang, Peng Fang, Tianyuan Jiang, Chongyang Ma, Qiuyun Zhang

**Affiliations:** Beijing Key Lab of TCM Collateral Disease Theory Research, School of Traditional Chinese Medicine, Capital Medical University, Beijing, China

## Abstract

Acute-on-chronic liver failure (ACLF) is a serious and complicated disease that threatens human health because its pathogenesis is unclear, and the outcome of the current therapies has been less than satisfactory. A national famous doctor of traditional Chinese medicine, Qian Ying, created the Jieduan-Niwan Formula (JDNW), based on his long-term clinical experience. However, despite the good clinical outcome, the biological mechanism by which it works is unknown. In the current study, we established an ACLF rat model by administering human serum albumin (HSA) combined with D-galactosamine (D-GalN) and lipopolysaccharide (LPS) to explore the potential mechanism of JDNW in treating ACLF. The rats were treated with JDNW by administration of the model substances and sacrificed after 4, 8, and 12 h. Then we divided the rats into normal group, model at 4 h, model at 8 h, model at 12 h, JDNW at 4 h, JDNW at 8 h, and JDNW at 12 h. Biochemical and histopathological examinations were performed to compare the rats in different groups. Compared with the ACLF model group, expression levels of alanine transaminase, aspartate aminotransferase, total bilirubin, and TNF-*α* and IL-6 proteins were reduced in the JDNW group at the corresponding time points, the survival rates of rats were increased, and the pathological condition of the liver was improved. In addition, JDNW treatment improved the ultrastructure of hepatocytes and mitochondria and decreased the hepatocyte apoptosis index. E2F1, P53, P73, Apaf-1, p14ARF, caspase-3, caspase-6, and caspase-7 levels in the JDNW group were distinctly lower than those in the untreated rats. Moreover, Bcl-2 and Mcl-1 levels increased. Thus, JDNW decreases ACLF-induced mortality in rats by modulating the E2F1-mediated intrinsic apoptotic pathway.

## 1. Introduction

Acute-on-chronic liver failure (ACLF) is a newly discovered clinical syndrome, characterized by early chronic liver disease or cirrhosis with organ failure. The 28-day mortality rate is high (50–90%) [1]. Predisposing factors to develop the syndrome may be intrahepatic or extrahepatic, and potential chronic liver injury may occur whether or not the patient develops cirrhosis [2]. Once cirrhosis has transitioned from the compensated to the decompensated period, the short-term survival is 3–5 years. At this point, it is recommended to evaluate liver transplantation, except in the presence of contraindications [3]. Despite advances in medical treatment, clinical management of ACLF remains limited and challenging. When medical treatment fails, liver transplantation is the only option to save the patient's life [4].

However, traditional Chinese medicine has shown its superiority over conventional ACLF treatments owing to its multitarget, integrity effects with few side effects. A multicenter and randomized controlled trial demonstrated that a combination of Chinese and Western Medicine is effective for the treatment of ACLF, with a low mortality rate and better efficacy [5]. The Jieduan-Niwan formula (JDNW), which originated from the national celebrated traditional Chinese medicine expert Qian Ying, had a significant impact on this disease. In a clinical study, about 110 selected patients were administered the combined treatment of JDNW and Western Medicine or the Western Medicine alone. The combined treatment showed superior results in reducing mortality and in improving liver function and other symptoms as compared to the Western Medicine control group [6].

Apoptosis is a mechanism of programmed cell death and is essential for organism development and tissue homeostasis. The occurrence and onset of liver failure are closely related to apoptosis and inflammation [7]. Our previous studies showed that JDNW formula could prolong liver survival time and ameliorate its injury, which might be related to reduced levels of inflammatory cytokines, apoptotic index, and liver cell proliferation [8–14]. JDNW could stimulate the compensatory proliferation of hepatocytes by regulating the expression of E2F1 [15]. However, the main aim of this study was to investigate whether JDNW could reduce liver apoptosis in a rat model of ACLF, and if this mechanism was mediated by the E2F1 intrinsic pathway.

## 2. Materials and Methods

### 2.1. Reagents

Human serum albumin (HSA, A9731-5G), D-Galactosamine (D-GalN, G0500-25G), and Lipopolysaccharide (LPS, 109K4075) were purchased from Sigma-Aldrich (St. Louis, MO, USA). Colorimetric TUNEL Apoptosis Assay Kit was purchased from Beyotime (C1098). ELISA kits for Rat IL-6 (CRE005) and Rat TNF-*α* (CRE003) were purchased from 4A Biotech Co., Ltd.

### 2.2. Preparation and High-Performance Liquid Chromatography (HPLC) Analysis of JDNW

**Figure 1:**
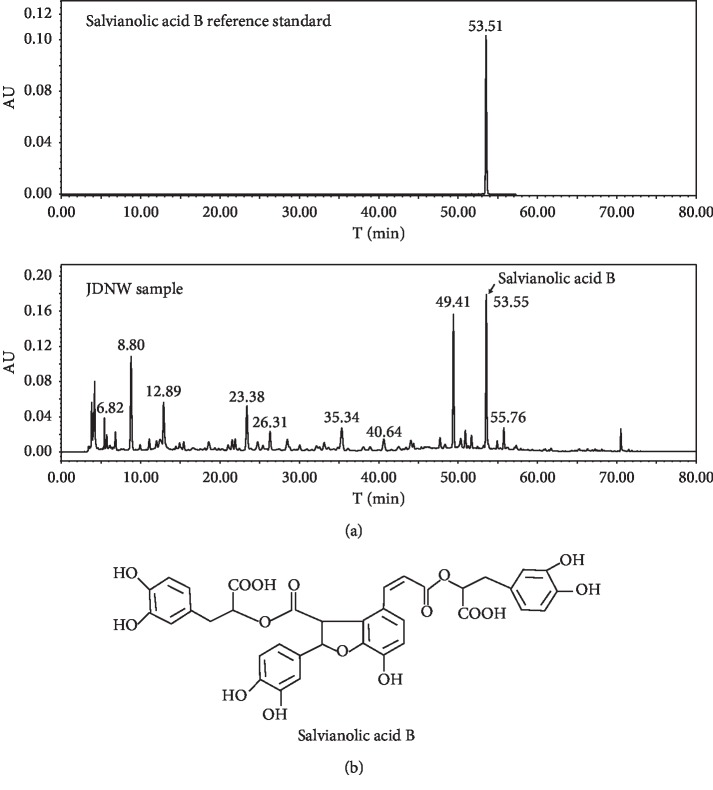
(a) The HPLC chromatograms of standard salvianolic acid B and JDNW extract. (b) Salvianolic acid B chemical structure.

DNW was prepared from ten commonly used herbs (Table 1): *Phyllanthus urinaria* Linn., Radix Astragali, Fructus Trichosanthis, Herba Lysimachiae, Herba Visci, Radix et Rhizoma Notoginseng, Rhizoma Curcumae, Radix et Rhizoma Salviae Miltiorrhizae, Radix Rehmanniae, and Radix Aconiti Lateralis Preparata. The raw herbs were purchased from the Beijing Tong Ren Tang Group Co. Ltd. (Beijing, China). They were mixed in the ratio of 30 : 30 : 30 : 30 : 30 : 6: 6 : 20 : 20 : 15 (dry weight in grams). All herbs were decocted twice. The two cycles of filtered suspensions were mixed and concentrated to 50 mL (with a density of 4.34 g/mL) with a vacuum rotary evaporator. Subsequently, the aqueous solution was stored at 4°C and heated to 37°C in a water bath before use. For quality control purposes, salvianolic acid B (the representative component of JDNW) was determined by HPLC. The results are shown in Figure 1.

### 2.3. Animal Model and Treatment

Male Wistar rats, weighing 180–200 g, were obtained from the Academy of Military Medical Sciences Laboratory Animal Center (Beijing, China). License number was SCXK-(Beijing) 2016–0006. Animals were housed in a specific pathogen-free environment. All protocols were approved by the Animal Experiments and Experimental Animal Welfare Committee of Capital Medical University (Beijing, China).

**Figure 2:**
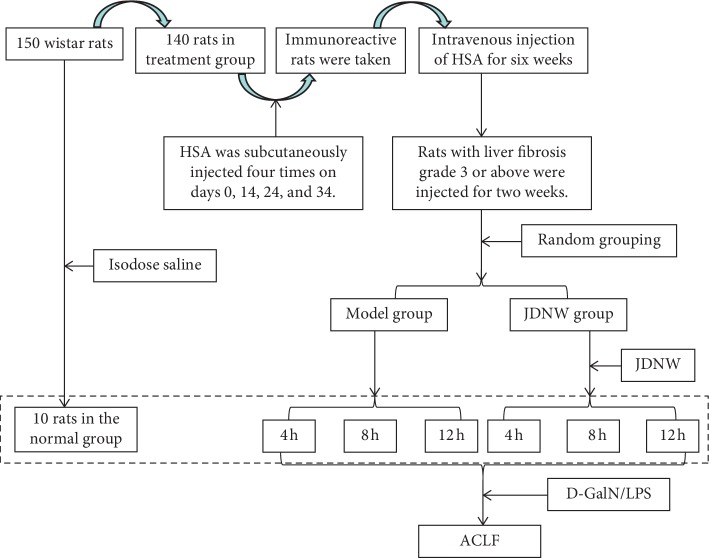
Experimental setup.

The ACLF rat model we used was established as described previously [16]. Briefly, ACLF was induced by combined administration of human serum albumin (HSA) with D-galactosamine (D-GalN) and lipopolysaccharide (LPS). Rats were divided into two model stages (Figure 2). (1) Liver cirrhosis model stage: the rats (*n* = 150) were randomized into two groups, the control group (*n* = 10) and the treatment group (*n* = 140). The former group was given multiple subcutaneous injections of 0.5 mL saline solution; meanwhile the latter one was injected with one dose of HSA (containing 4 mg) for sensitization, followed by administration of 0.5 mL HSA into the tail vein twice a week. Six weeks later, the successful establishment of models was confirmed by Masson's trichrome staining (see Supplementary [Supplementary-material supplementary-material-1]). Finally, 98 liver cirrhosis model rats were randomly divided into ACLF model and JDNW groups with 49 rats in each group. (2) ACLF model stage: model and JDNW groups were given acute attack induced by simultaneous administration of 400 mg/kg D-GalN and 100 *μ*g/kg LPS into the enterocoelia. Rats in the control group received the same volume of saline solution. On the other hand, rats of the JDNW group were administered 21.7 g/kg/d JDNW decoction via oral gavage twice a day, for 3 days, which was the optimized dosage-regimen according to our preliminary study [17].

Each group of rats was randomly divided into 3 subgroups and sacrificed at 4, 8, and 12 h, respectively, following D-GalN and LPS administration. After intraperitoneal injection of 3% sodium pentobarbital anesthetic (30 mg/kg), blood was collected from the aorta and used for biochemical analyses. Furthermore, the liver was removed, and the right lobe was fixed in 10% neutral formalin buffer solution and embedded in paraffin for pathological staining. Additionally, a small portion of liver tissue from the ipsilateral side was fixed with 2.5% glutaraldehyde solution for electron microscopy, and other lobes were dissected and frozen in liquid nitrogen for further proteomic study.

### 2.4. Blood Sample Examination

Blood was centrifuged for 15 min at 4°C and 3000 rpm and serum was collected. The serum levels of alanine transaminase (ALT), aspartate transaminase (AST), and total bilirubin (TBIL) were detected by using a Hitachi 7600 Automatic Analyzer (Hitachi, Inc., Japan).

### 2.5. ELISA of the Hepatic Tissue

The liver tissue was homogenized using 10 mL of PBS for 1 g of tissue and centrifuged at 4500 rpm for 5 min to obtain the supernatant. The concentrations of TNF-*α* and IL-6 in the liver tissue were detected by ELISA according to the kit instructions.

### 2.6. Survival Rate

A 24-hour survival group comprising 21 rats was randomly divided into 3 groups: (1) control group, *n* = 7; (2) disease model group, *n* = 7; and (3) JDNW group, *n* = 7. Survival rates were measured at 24 h after administration of combined HSA, LPS, and D-GalN.

### 2.7. Histological Observation of the Liver

Embedded liver tissues were sliced and stained with hematoxylin-eosin (HE). The stained samples were observed with a Nikon Eclipse 80i microscope at ×200 magnification.

### 2.8. TUNEL Assays

TUNEL assay was performed according to the manufacturer's protocol (Beyotime). Apoptosis was observed with a Nikon Eclipse 80i microscope at ×200 magnification. According to the distribution of apoptotic cells, 5 areas were analyzed per section, and 100 cells were counted in each area. The apoptotic index (AI) was calculated as the percentage of the number of apoptotic cells/total number of cells.

### 2.9. Transmission Electron Microscopy (TEM) Observation

Liver specimens were fixed with 2.5% glutaraldehyde for 2 h and washed three times for 10 min each with phosphate buffer. After dehydration with ethanol and propanol, liver specimens were embedded in epoxy resin sliced and stained with uranium acetate and lead citrate. The ultrastructure of hepatocytes and mitochondria was observed and photographed by transmission electron microscopy.

### 2.10. Western Blot Analysis

Liver tissues (50–100 mg) were harvested and homogenized from 6 rats in each group. The protein concentration in each sample was determined according to the kit instructions. An aliquot of 30 *μ*g from each sample was separated by 12% SDS-PAGE for 1 h at 120 V. Bands were electrophoretically transferred to 0.22 *μ*m PVDF membranes (Millipore, USA), then blocked for nonspecific binding with 5% nonfat dry milk for 1 h, and incubated overnight at 4°C with primary antibodies. The next day, after washing, the membranes were incubated with Goat Anti-Mouse IgG and Goat Anti-Rabbit IgG (Santa Cruz Biotechnology, USA) for 1 h reaction, washed again, and developed with an electrochemiluminescence (ECL) reagent (Millipore, USA). Blots were developed by autoradiography. *β*-Actin antibody (Abcam, USA, 1 : 3000) was used as an internal control. Images were analyzed and quantified by using ImageJ software. The following primary antibodies were used: E2F-1 antibody (Santa Cruz Biotechnology, USA, 1 : 500), P53 antibody (Santa Cruz Biotechnology, USA, 1 : 1000), P73 antibody (Abcam, USA, 1 : 1000), Apaf-1 antibody (Santa Cruz Biotechnology, USA, 1 : 1000), caspase-3 antibody (CST, USA, 1 : 1000), caspase-6 antibody (Proteintech, 1 : 500), caspase-7 antibody (Santa, USA, 1 : 1000), p14ARF antibody (Abcam, USA, 1 : 500), Bcl-2 antibody (Santa Cruz Biotechnology, USA, 1 : 1000), and Mcl-1 antibody (Abcam, USA, 1 : 1000).

### 2.11. PCR

Total RNA was extracted from rat liver samples using TRIzol reagent and its concentration and purity were determined using a spectrophotometer. RNA samples with A260/A280 from 1.8 to 2.2 were selected and their integrity was tested on 2% agarose gels. The total RNA was then reverse-transcribed into cDNA using PrimeScript™ RT reagent Kit with gDNA Eraser. PCR primers were designed and synthesized by Invitrogen; the primer sequences have been provided in Supplementary Material ([Supplementary-material supplementary-material-1]). Finally, real-time PCR was performed on target genes and the data were analyzed by 2^−ΔΔCt^ method.

### 2.12. Statistical Analyses

Statistical analyses were performed by using SPSS software version 19.0. Experiments were repeated at least three times and results presented as mean ± standard deviation (SD). One-way ANOVA was used to compare the significance of the differences among groups, the least-significant difference (LSD) method was applied when the variance was equal among groups, and Tamhane's T2 test was performed otherwise. *P* values <0.05 were considered to be statistically significant.

## 3. Results

### 3.1. Serum ALT, AST, and TBIL Levels and Survival Analysis

**Figure 3:**
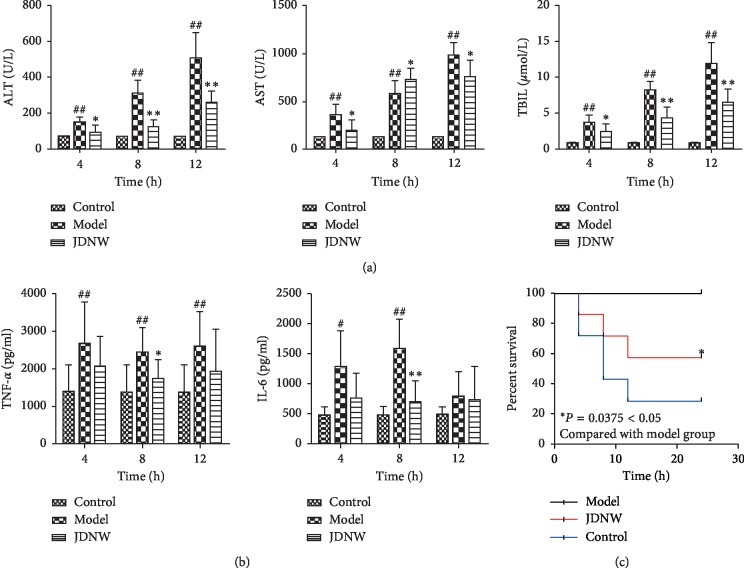
Rat liver functions, assessed by levels of parameters, inflammatory factors, and survival rates. (a) Levels of ALT, AST, and TBIL. (b) Levels of TNF-*α* and IL-6. (c) ACLF rat survival curves within 24 hours. ^#^*P* < 0.05; ^##^*P* < 0.01, versus control group. ^*∗*^*P* < 0.05; ^*∗∗*^*P* < 0.01, versus model group.

Figure 3(a) shows the analysis of liver function parameters. Compared to the control group, ALT, AST, and TBIL levels in the ACLF model group were significantly and continuously increased through time (*P* < 0.01), which indicated the presence of severe liver damage. However, the JDNW group showed reduced ALT, AST, and TBIL serum levels. Differences were statistically significant at 8 and 12 h (*P* < 0.01). The Kaplan-Meier survival curves (Figure 3(c)) showed that the 24-hour survival rate of JDNW group was significantly increased compared to that of the ACLF model group (*P* < 0.05). After 24 hours, only 2 rats survived (survival rate = 28%) in the model group, whereas 4 rats survived (survival rate = 57%) in the JDNW group. Overall, the survival time of the JDNW group was longer than that of the model group (*P* < 0.05) (Table 2).

### 3.2. ELISA

Levels of TNF-*α* and IL-6 proteins (Figure 3(b)) were remarkably increased in the ACLF model group; however, treatment with JDNW significantly reduced their levels at 8 h (TNF-*α*: *P* < 0.05; IL-6: *P* < 0.01).

### 3.3. Liver Histology Observation

**Figure 4:**
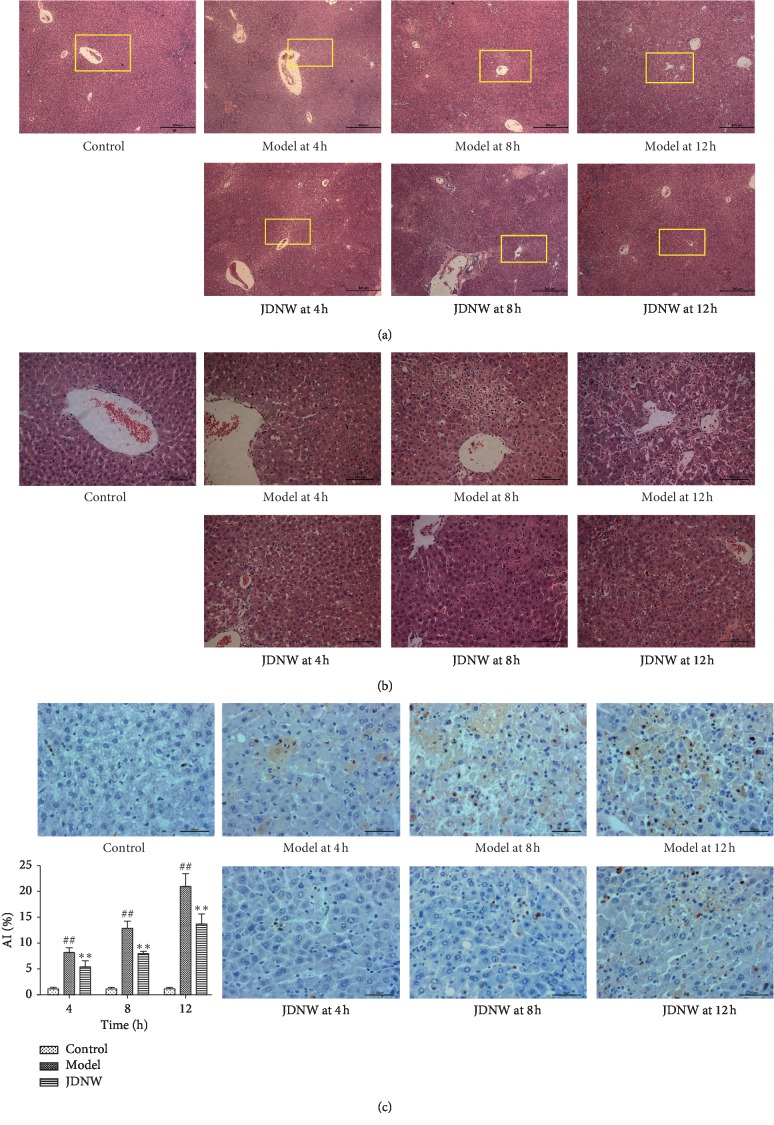
The histological changes in each group were observed after HE staining. (a) Magnification ×50. (b) Magnification ×200. (c) TUNEL assay results at different time points. ^#^*P* < 0.05; ^##^*P* < 0.01, versus control group. ^*∗*^*P* < 0.05; ^*∗∗*^*P* < 0.01, versus model group.

A representative picture of HE-stained liver tissue for each group is shown in Figures 4(a) and 4(b). In the control group, liver cells were arrayed around the central vein and neither degeneration nor necrosis was presented. In the ACLF model group, hepatocyte swelling, apoptotic body formation, inflammatory cell infiltration, internal bleeding, disorganized liver cell arrangement, and massive or submassive necrosis were revealed in some areas and the severity increased over time. However, the histological characteristics of the liver were significantly improved at each corresponding time point in the JDNW group.

### 3.4. TUNEL Assay Results

As shown in Figure 4(c), apoptotic cells were detected in the control group. On the contrary, a large number of apoptotic cells with brown nucleus appeared at 4, 8, and 12 h and gradually increased over time in the model group. However, apoptotic cells in the JDNW group decreased at the corresponding time points. These results suggested that JDNW could alleviate hepatocyte apoptosis and reduce the apoptotic index.

### 3.5. Ultrastructure Observation

**Figure 5:**
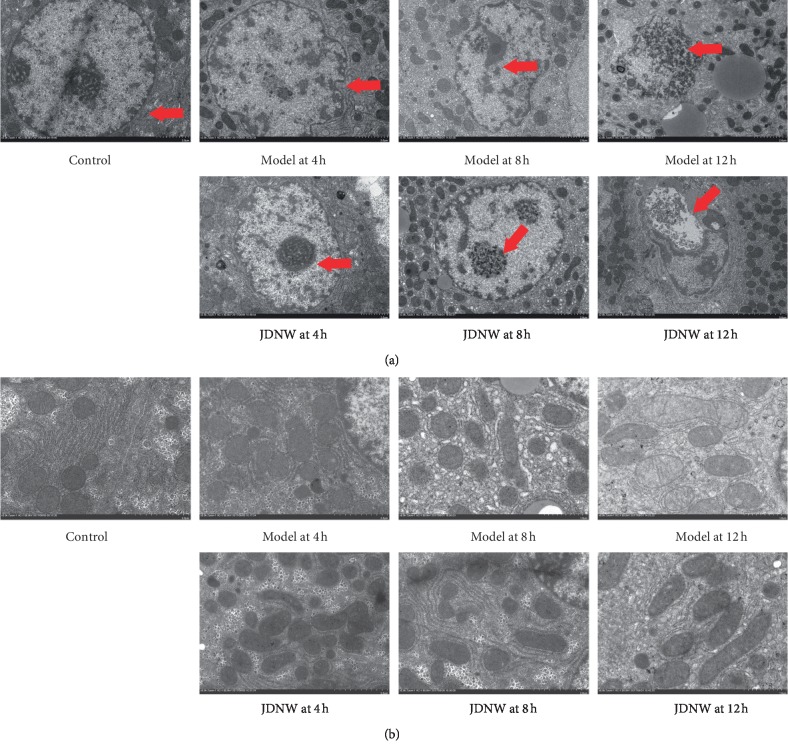
(a) Ultrastructure changes of rat hepatocytes in the rats of each group (bar = 2 *μ*m). (b) Ultrastructure changes of mitochondria in the rat hepatocytes of the rats in each group (bar = 500 nm).

In the control group, the hepatocyte membrane (Figure 5(a)) was intact, the cytoplasm was homogeneous, and the nucleus was round and centered. The shape of mitochondria (Figure 5(b)) was round, and the structures of the cristae and bilayer membrane were intact. In the 4-hour model group, nuclear deformation and chromatin condensation were observed. Also, cytoplasm became gradually empty; meanwhile, mitochondria presented a swelling, dumbbell appearance. In the 12-hour model group, irregular or ruptured hepatocytes nuclei were observed; meanwhile, Kupffer cells invaded necrotic cells and engulfed apoptotic bodies. Comparing with the model group, the ultrastructures of hepatocytes and mitochondria in the JDNW group were significantly improved at each corresponding time point.

### 3.6. Western Blotting and PCR Analysis

**Figure 6:**
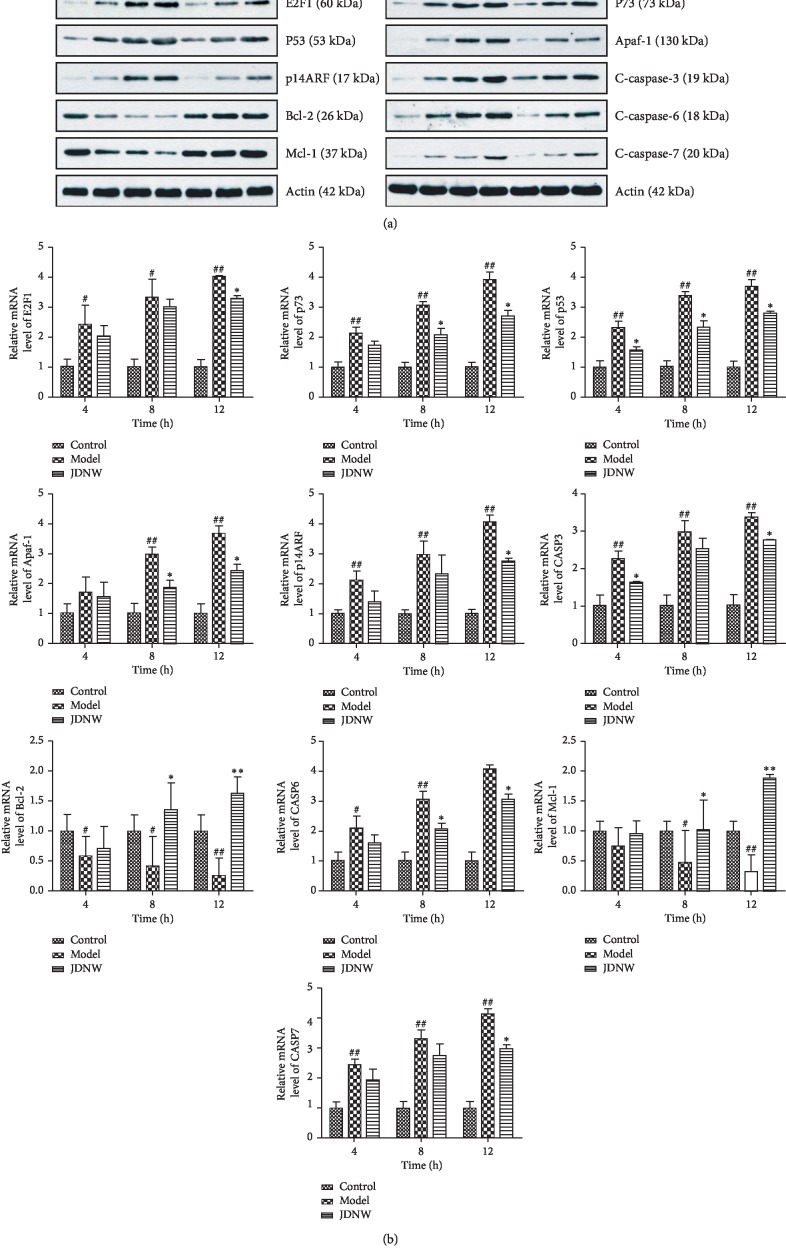
Effect of JDNW on expression of E2F1, P53, P73, p14ARF, Apaf-1, caspase-3, caspase-6, caspase-7, Bcl-2, and Mcl-1 levels in liver tissues of ACLF model rats. (a) Western blot analysis. A: control. B: model at 4 h. C: model at 8 h. D: model at 12 h. E: JDNW at 4 h. F: JDNW at 8 h. G: JDNW at 12 h. (b) mRNA expression. ^#^*P* < 0.05; ^##^*P* < 0.01, versus control group. ^*∗*^*P* < 0.05; ^*∗∗*^*P* < 0.01, versus model group.

As compared with the control group, expression levels of E2F1, P53, p14ARF, P73, Apaf-1, and cleaved caspase-3, caspase-6, and caspase-7 were increased, whereas those of Bcl-2 and Mcl-1 were decreased in the ACLF model group (Figures 6(a) and 6(b)). Treatment with JDNW reduced E2F1, P53, p14ARF, P73, Apaf-1, and cleaved caspase-3, caspase-6, and caspase-7 levels but increased Bcl-2 and Mcl-1 levels (protein expression data have been provided in the supplementary data of this manuscript).

## 4. Discussion

ACLF is a syndrome characterized by acute jaundice and coagulation dysfunction. It can be combined with complications such as hepatic encephalopathy, ascites, electrolyte disturbance, infection, hepatorenal syndrome, hepatopulmonary syndrome, extrahepatic organ failure, and new hepatocyte necrosis lesions of varying degrees [18]. Administration of LPS and D-GalN is a commonly used approach to generate a rat model of ALF [19]; however, only two different methods have been used to induce the basic chronic liver injury: CCl_4_ and HSA [20–22]. When compared with the hepatotoxic drug CCl_4_, the immunologic stimulant HSA can induce a more stable model with lower healing rate, and a pathogenetic mechanism more similar to that in ACLF [23, 24]. Therefore, we used a combined administration of HAS, LPS, and D-GalN to generate the animal model in the study.

Disturbance and necrosis of hepatocytes and elevated levels of ASL and ALT suggested liver failure [25]. Our results showed that, ALT, AST, and TBIL protein levels increased in the ACLF model group. In addition, histopathology analysis showed massive or submassive necrosis and collapse of parenchymal, TEM examination detected nuclear fragmentation, vacuolization of mitochondria, and apoptotic bodies formation, which indicated that the ACLF model was successfully established. After JDNW intervention, liver function improved, the apoptotic index decreased, and survival times were prolonged.

JDNW, originally created by Qian Ying professor, has been used in Beijing You'an Hospital, Capital Medical University, for many years. Previous clinical trials have also confirmed the protective effects of JDNW on ACLF patients [7]. The drug contained ten Chinese herb medicines, among them, *Phyllanthus urinaria* Linn., Fructus Trichosanthis, Herba Lysimachiae, Radix et Rhizoma Notoginseng alleviated heat, induced disintoxication, resolved stasis, and eliminated jaundice. Also, Radix Astragali and Herba Visci played the role of tonifying spleen and nourishing the liver and kidney. Rhizoma Curcumae and *Salvia miltiorrhiza* Bunge played the role of resolving toxin and dredging collaterals. Radix Rehmanniae and Radix Aconiti Lateralis Preparata regulated Yin and Yang.

However, abstract TCM theory is not able to fully explain the mechanism of JDNW in treating liver failure. LPS- and D-GalN-induced liver failure is associated with oxidative stress, inflammatory response, and apoptosis [26]. E2F1 is an activator of transcription and has the function of regulating apoptosis and proliferation [27]. In addition, E2F-1 had the ability to induce apoptosis in many cells [28, 29, 30, 31]. Based on this, we aimed to study whether JDNW regulates E2F1 pathway. In the p53-dependent pathway, E2F-1 expression induced apoptosis through the accumulation of p53 and thus by activating p14ARF expression [32]. In our study, E2F1, p53, and p14ARF levels gradually increased in the model group but decreased in the JDNW group. The results suggested that transcription factors were activated, and JDNW had an antiapoptotic effect. In the p53-independent apoptosis pathway, E2F1 directly activated some apoptotic genes, such as p73, the homologous gene of p53 [33], and Apoptotic protease activating factor 1 (Apaf-1) [34], thereby activating the proapoptotic effector enzymes caspase-3, caspase-6, and caspase-7 and inducing apoptosis [35]. Protein expression of P73 and Apaf-1 in the model group, and of their downstream genes, caspase-3, caspase-6, and caspase-7 increased in our study. When apoptosis occurs, caspase-3, caspase-6, and caspase-7 in the proenzyme forms are cleaved into cleaved caspase-3, caspase-6, and caspase-7, respectively. Apaf-1 is the core component of apoptosis body and plays an important role in the mitochondrial apoptotic pathway [36]. Besides protecting mitochondrial morphology, JDNW also reduced the expression of P73 and Apaf-1 and inhibited the activation of caspase-3, caspase-6, and caspase-7 and thus inhibited apoptosis. E2F1 also blocks antiapoptotic pathway and induces apoptosis by inhibiting Bcl-2 and Mcl-1 [37], which are antiapoptotic members of the Bcl-2 family. E2F-1 inhibited Bcl-2 and Mcl-1 expression and induced apoptosis in the model group. Compared to the model group, Bcl-2 and Mcl-1 expression increased in the JDNW group at corresponding time points, which suggested that JDNW exerted an antiapoptotic effect.

Serum TNF-*α* and IL-6 are more sensitive than ALT and AST levels in predicting liver injury [30]. TNF-*α* is a primary and central inflammatory cytokine that induces hepatocyte apoptosis and liver injury [31]. IL-6 plays a role as a proinflammatory cytokine in chronic inflammatory disease models [38, 39]. Additionally, acute inflammatory IL-6 driven response might contribute to the transition from a stable chronic state to a progressive liver injury [40]. Following treatment with JDNW, the expression of inflammatory cytokines was inhibited. Therefore, the effect of ACLF on the E2F1 mediated intrinsic apoptosis pathway was mainly due to the *in vivo* increase of endotoxins, which promoted the secretion of inflammatory cytokines, such as TNF-*α* and IL-6, causing apoptosis.

ACLF rat model was successfully reproduced and our results suggested that JDNW could inhibit liver failure by inhibiting hepatocyte apoptosis. However, the time required to induce ACLF is longer and the process is more complex. Therefore, simpler methods need to be urgently explored. Moreover, there are too many targets of TCM; whether JDNW alone acts on E2F1 and then changes downstream proteins remains to be confirmed. Network pharmacology has advantages in analyzing the relationship between Chinese herbal compound and target [41]. Therefore, the next step is to combine network pharmacology with *in vitro* experiments and to add E2F1 inhibitors to confirm the role of JDNW.

## 5. Conclusions

**Figure 7:**
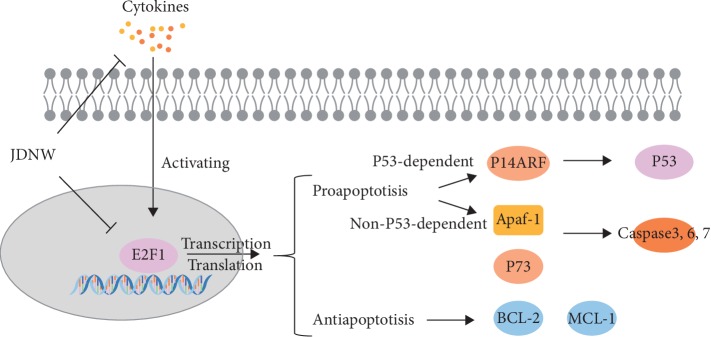
The mechanism of E2F1-induced apoptosis, and inhibition of this pathway by JDNW in ACLF rats.

In summary, this study suggested that JDNW could significantly protect rat liver and attenuate hepatocyte apoptosis in ACLF rats. We also showed that this effect was due to inhibition of the E2F1-mediated intrinsic apoptosis pathway. Therefore, we propose a therapeutic pathway through which JDNW could reduce liver apoptosis in ACLF (Figure 7).

## Figures and Tables

**Table 1:** Composition of JDNW.

Components	Amount used (g)	Botanical name of plant	Part used	Family
Phyllanthus urinaria Linn.	30	*Phyllanthus amarus Schumach*. *& Thonn*	Herb	Euphorbiaceae
Radix Astragali	30	*Astragalus membranaceus (Fisch*.*) Bunge*	Root	Leguminosae
Fructus Trichosanthis	30	*Trichosanthes kirilowii Maxim*	Fruit	Cucurbit
Herba Lysimachiae	30	*Lysimachia christinae Hance*	Herb	Primulaceae
Herba Visci	30	*Viscum coloratum (Kom*.*) Nakai*	Stem, leaf	Loranthaceae
Radix et Rhizoma Notoginseng	6	*Panax notoginseng* (Burkill) F. H. Chen	Root	Araliaceae
Rhizoma Curcumae	6	*Curcuma phaeocaulis* Valeton	Rhizome	Zingiberaceae
*Salvia miltiorrhiza* Bunge	20	*Salvia miltiorrhiza Bunge*	Root	Lamiaceae
Radix Rehmanniae	20	*Rehmannia glutinosa* (Gaertn.) DC	Root	Scrophulariaceae
Radix Aconiti Lateralis Preparata	15	*Aconitum carmichaeli var. carmichaeli*	Root	Ranunculaceae

**Table 2:** Comparison of survival time of rats in each group.

Groups	N	Survival periods (h)
Model	7	12.00 ± 8.64
JDNW	7	17.14 ± 8.86^*∗*^

^*∗*^ *P* < 0.05 versus model group.

## Data Availability

The data used to support the findings of this study are available from the corresponding author upon request.

## Supplementary Materials

Supplementary Materials (1) Masson staining of cirrhosis. (2) Primer sequence used for real-time PCR. (3) Protein expression data for each protein.
