# Supplementary material for: Jieduan-Niwan Formula Reduces Liver Apoptosis in a Rat Model of Acute-on-Chronic Liver Failure by Regulating the E2F1-Mediated Intrinsic Apoptosis Pathway
Source: Evid Based Complement Alternat Med. 2019 Nov 11;2019:8108503. doi: 10.1155/2019/8108503 (PMC6885299; doi:10.1155/2019/8108503)
Supplement: Supplementary Materials — (1) Masson staining of cirrhosis. (2) Primer sequence used for real-time PCR. (3) Protein expression data for each protein. [file 8108503.f1.pdf]

Supplementary material.

1. To confirm the successful development of liver cirrhosis, we performed Masson's trichrome staining of liver biopsy. It showed that collagen fibers formed fibrous septa and disordered lobular structure.

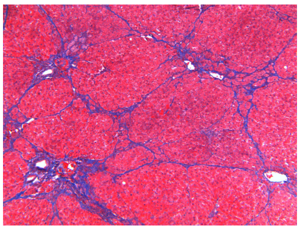

Figure 1: Masson's trichrome staining in liver cirrhosis model group (magnification ×200).

2. Primer sequence used for real-time PCR.

Table 1: Primer sequence used in real-time PCR detection and analysis.

Gene symbol	Primers	Amplicon (bp)
casp3	F:ACTGGAAAGCCGAAACTCTTCATCA	127
	R: GGAAGTCGGCCTCCACTGGTATC	
p53	F: TCACCTCCACACCTCCACCT	188
	R: CCTGTCGTCCAGATACTCAGCATA	
casp7	F: TATCAACGACACCGACGCTAAT	82
	R: GCCTGGAACCGTGGAGTAAG	
P14ARF	F: GCTCTCCTGCTCTCCTATGGT	269
	R: CAGAAGTTATGCCTGTCGGTGA	
Bcl-2	F: GGGCTACGAGTGGGATACTGGAG	101
	R: CGGGCGTTCGGTTGCTCT	
E2F1	F: AACGGAGGCTGGATCTGGAA	293
	R: ATTCTTGGACTTCTTGGCAATGAG	
Apaf-1	F: GCTCTCCTCTCGTCGTGTCT	200
	R: GTCTGTGTAGTAGTCCTTGATGTCT	
MCL1	F: GCCAAACACTTAAAGAGCATAAACC	87
	R: CCGCTTCGTCCTTACAAGAAC	
Casp6	F: AGAAGAACTACTGCTCAAGATTCAC	180
	R: CCAACCAGGCTCTGACACTT	
P73	F: TGTCGCCAACCTTATCAAGCTC	139
	R: GCATGGCCAGCTCTGTTCTC	
PCNA	F: CATATTGGAGATGCTGTGGTGAT	231
	R: CATACTGAGTGTTACTGTAGGAGAC	
Actin	F: GGAGATTACTGCCCTGGCTCCTA	150

R: GACTCATCGTACTCCTGCTTGCTG

3. Protein expression data for each protein.

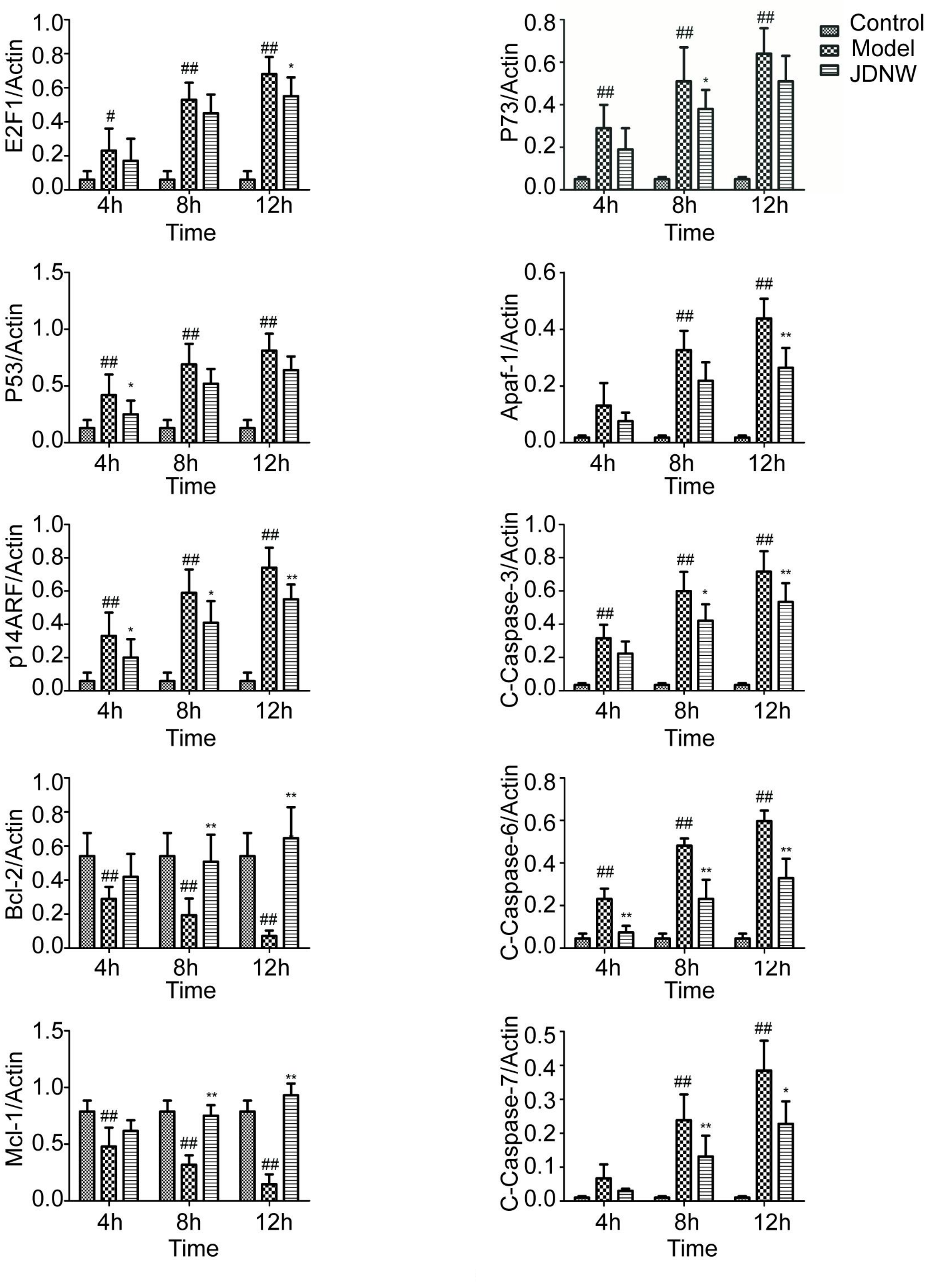

Figure 2: Effect of JDNW on the expression of E2F1, P53, P73, p14ARF, Apaf-1, Cleaved Caspase-3, 6 and 7, and Bcl-2 and Mcl-1 protein expression levels in the liver tissues of ACLF model rats. $^{\#}P<0.05$; $^{\#\#}P<0.01$, vs. control group. $^{*}P<0.05$; $^{**}P<0.01$, vs. model group.
